# The dual functions of Ziziphi spinosae semen as medicine and food: origins, cross-domain applications, antidepressant metabolites, and pharmacological mechanisms

**DOI:** 10.3389/fphar.2026.1735895

**Published:** 2026-04-20

**Authors:** Xia Shao, Mingzhe Huangfu, Ning Wang, Qi Zhang, Minghui Zhao, Wanqing Xie, Chengbo Zhang, Ting Ma

**Affiliations:** 1 School of Rehabilitation Medicine, Shandong University of Traditional Chinese Medicine, Jinan, China; 2 China Academy of Chinese Medical Sciences, Hospital of Guang’anmen Hospital, Jinan, China; 3 Institute of Chinese Medicinal Literature and Culture, Shandong University of Traditional Chinese Medicine, Jinan, China; 4 Research Department of Shandong University of Traditional Chinese Medicine, Jinan, China

**Keywords:** application, bioactive metabolites, depression, ethnopharmacology, ziziphi spinosae semen

## Abstract

Depression, a serious mental illness prevalent globally, exhibits a strong clinical heterogeneity. Existing chemical drug treatments have significant limitations, necessitating the development of safe and effective new intervention strategies. *Ziziphi Spinosae Semen*, included in the first group of medicinal botanical drugs in China’s “dual functions of medicine and food” database, combines traditional sedative effects with modern pharmacological activities, demonstrating unique potential for the prevention and treatment of depression. This article systematically reviews literature from databases such as PubMed, Web of Science, Google Scholar, CNKI, and Wanfang Data. It elucidates the significance of its “dual functions of medicine and food” by tracing its journey from traditional use in prescriptions to modern food and health applications. The review focuses on summarizing its four major categories of antidepressant bioactive metabolites and deeply analyzes its multi-dimensional synergistic mechanisms of action. These include regulating monoamine neurotransmitter homeostasis, repairing hypothalamic-pituitary-adrenal axis hyperfunction, upregulating brain-derived neurotrophic factor expression, and inhibiting neuroinflammatory responses. This synthesis aims to provide new insights and a scientific basis for developing novel antidepressant drugs or functional dietary supplements based on the “dual functions of medicine and food” concept.

## Graphical Abstract

**Figure:**
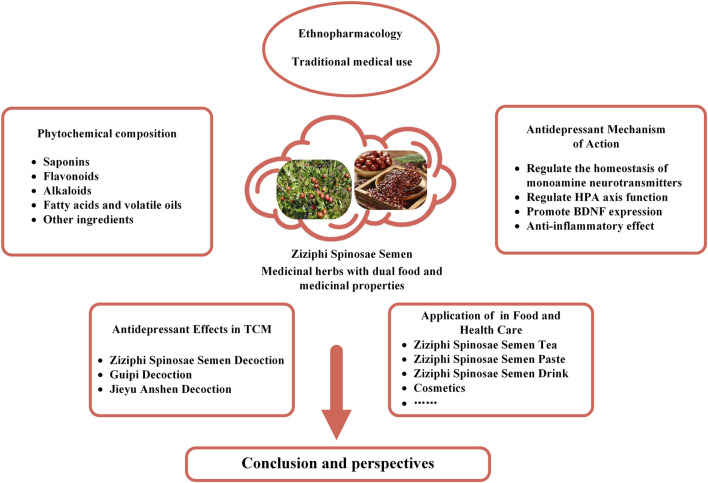

## 1 Introduction

**FIGURE 1:**
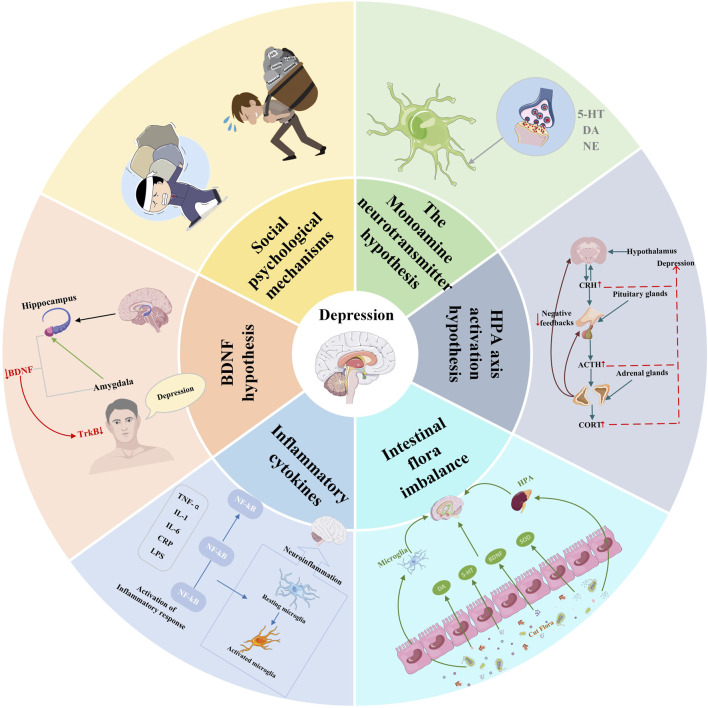
Hypothesis of the pathogenesis of depression.

Depression is a highly heterogeneous mood condition defined by enduring poor mood, anhedonia, and hopelessness, accompanied by anxiety, insomnia, slowed reaction times, and impaired learning and memory abilities. Severe instances may result in suicidal inclinations (McCarron et al., 2021). According to the WHO, depression affects over 350 million people worldwide. Over 75% of patients experience recurrent depressive symptoms throughout their lives. By 2030, depression is anticipated to become the primary contributor to the global disease burden (Liu Q et al., 2020). Due to the wide interindividual variability in clinical manifestations and treatment responses, and the unpredictable course and prognosis of depression, clinical diagnosis and treatment present significant challenges, posing a serious threat to public health and increasing the global burden of disease. Currently, traditional Western medicine antidepressants, such as SSRIs and TCAs, are the most common treatment options (Duman et al., 2019). However, these antidepressants have a limited mode of action, are prone to numerous adverse reactions, are expensive, require long treatment cycles, and have a high relapse rate after discontinuation (Zhang et al., 2024; Henssler et al., 2024; Rennwald and Hengartner, 2025). The pathogenesis of depression is complex and is influenced by multiple factors such as social psychology and neurophysiology. Representative hypotheses include reduced monoamine neurotransmitters, HPA axis dysfunction, imbalance in the expression of brain-derived neurotrophic factor, increased inflammation levels, and gut microbiota imbalance (Figure 1).

In recent years, with the continuous promotion of the “Big Food Concept” and the “Healthy China” strategy, the integration of traditional medicine and modern nutrition has deepened and the concept of dual functions as food and medicine has become an increasingly important direction in academic research and health practice. The Yellow Emperor’s Classic of Internal Medicine asserts, “If used to satisfy hunger, it is called food; if used to treat illness, it is called medicine.” While providing satiety, they can also boost the body’s immune system, playing a preventive and therapeutic role (Gong et al., 2020; Xue et al., 2022; Lu et al., 2023). For example, foods like *Zingiber*, *Allium*, *Ziziphus jujuba*, and *Panax ginseng* have been used for centuries not only as part of daily diets but also for their medicinal properties to address various health issues (Danxi et al., 2025). To date, the National Health Commission has published over 100 Chinese medicinal botanical drugs with the dual functions of food and medicine (Zhou et al., 2025). An increasing volume of research suggests that numerous Chinese botanical drugs, sharing dual functions as food and medicine, can provide antidepressant effects via modulating neurotransmitter metabolism and receptor expression, regulating the HPA axis, and improving gut microbial metabolism (Yu, 2024; Tian et al., 2023; Kou et al., 2025).

*Ziziphus jujuba Mill. var. spinosa (Bunge) Hu ex H.F. Chow* (also known as *Ziziphi Spinosae Semen*, ZSS) is a commonly used traditional Chinese medicinal botanical drug in China and one of the first to be included in the list of medicinal and edible botanical drugs. They exhibit a range of biological activities, encompassing calming and hypnotic effects, anti-anxiety and depression, anticonvulsant properties, cardiovascular protection, anti-inflammatory properties, and improved learning and memory (Table 1). They are widely used in the treatment of clinical emotional disorders and in various health supplements. Studies have shown that ZSS can be used medicinally to treat insomnia, anxiety, and depression, and can also be used as a dietary supplement for daily wellbeing. For example, they can be combined with *Dimocarpus longan* and *Smilax glabra* to create a medicinal porridge, or brewed with *Lilium lancifolium* and *Nelumbo nucifera* to create a calming tea. Multiple studies have validated that ZSS encompass an array of bioactive metabolites, such as flavonoids, saponins, and alkaloids, and is extensively utilized in the prophylaxis and management of depression (Du.C.H et al., 2022; Li et al., 2022). Therefore, guided by the TCM theory of “dual functions as food and medicine,” this article systematically examines the origins and bioactive metabolites of ZSS in preventing and treating depression, as well as their antidepressant effects and their underlying mechanisms. This aims to clarify their pharmacological properties and offer theoretical support and a reference for the development and practical application of novel antidepressant medicines derived from ZSS.

**TABLE 1:** Pharmacological effects of ZSS.

Pharmacological effect	Activity/Mechanism(s) of action	Cell lines/Model	Dose	Application	References
Antidepressant	Blocking the PI3K/Akt and MAPK/ERK signal transduction pathways; dephosphorylates the CREB.	Mice LLC lung cancer cells and human A549 cells; C57BL/6 mice	60 mol/L; 40 mg/kg	*In vitro* and *vivo*	Yang et al. (2022)
	Increase the levels of NE, DA, and 5-HT in prefrontal cortex	ICR mice	15 mg/kg	*In vivo*	Li et al. (2022c)
	Downregulates IL-6 expression, upregulates IL-10 and the expression of Fizzl and ARG1 proteins in the rat hippocampus	SD rats	2.475, 4.95, 9.9 g/kg	*In vivo*	Li J et al. (2025)
	Inhibition of TLR4/NF-κB/NLRP3 signaling pathway activation	KM mouse	50, 100, 150 mg/kg	*In vivo*	Zhang J et al. (2025)
	Regulation of FAM19A5, SYN, PSD-95, IL-10, IL-6, and SP1/SK1/S1PR1 expression in the hippocampus	SD rats	10, 5, 2.5 g/kg	*In vivo*	Feng et al. (2025)
	Increases 5-HT levels, inhibits NLRP3 signaling pathway activation, and reduces the levels of inflammatory factors	SD rats	1.8, 3.6, 7.2 g/kg	*In vivo*	Zhang Y et al. (2025)
	Activation of the PI3K/AKT signaling pathway inhibits excessive ERS in cells and reduces neuronal apoptosis	PC12 cells; SD rats	4, 16 g/kg	*In vitro* and *vivo*	Huang C et al. (2025)
	Inhibiting the p38MAPK hippocampal pathway signaling reduces the level of the inflammatory mediator TNF-α	SD rats	2.475, 4.95, 9.9 g/kg	*In vivo*	Hao et al. (2025)
	Reduce the levels of brain inflammatory factors IL-6 and TNF-α	SD rats	3.24, 6.48, 12.96 g/kg	*In vivo*	Ma R et al. (2025)
	Regulation of *lactobacillus*, fecal bacteria, acetic acid, propionic acid, butyric acid, alanine, aspartate, and glutamate metabolic pathways	SD rats	12, 24 g/kg	*In vivo*	Chang et al. (2025)
	Regulating the 5-HT anabolism and HPA axis levels, related pathological damage in the hippocampus, hormone reducing improving synaptic plasticity, repairing BBB integrity, and alleviating inflammatory response and oxidative stress damage	C57BL/6J mice	1.95, 3.9, 7.8 g/kg	*In vivo*	Cheng et al. (2025)
​	Regulates JAK2/STAT3 and ERK1/2/CREB pathways, improves neuroinflammation, modulates HPA axis disorder, and increases 5-HT and BDNF levels	C57BL/6 mice	3, 6, 12 g/kg	*In vivo*	Zhao et al. (2024)
	Regulation of JNK/c-Myc/p53 expression in the hippocampus	SD rats	2.5, 5, 10 g/kg	*In vivo*	Du et al. (2025)
	Regulation of the endoplasmic reticulum stress IRE1α/ASK1/JNK pathway	SD rats	16, 8, 4 g/kg	*In vivo*	Shi et al. (2024)
	Upregulates the expression of synaptic plasticity-related proteins such as BDNF, TrkB, CREB, PSD95, and Beclin1	C57BL/6 mice	10, 30 mg/kg	*In vivo*	Li et al. (2024)
Anti-inflammatory	Inhibits the release levels of NO, TNF- α, and IL-1β, and suppresses intracellular ROS levels	BV-2 cells and HT-22 cells	2.5, 5, 10 μmol/L	*In vitro*	Zhang et al. (2018)
	Inhibit the expression of IL-1β, IL-6, TNF- α, ESR1, PI3KCA, PPARG mRNA and ESR1 protein	bMECs	50, 100, 150 μg/mL	*In vitro*	Wang et al. (2026)
	Reduce the activity of NF-κB and P-ERK1/2 signaling molecules and decrease the production of IL-6	SD rats	4.95, 9.9, 2.475 g/kg	*In vitro*	Huang et al. (2025)
	Regulates the levels of key lipid metabolites such as PA, PE, PC, LysoPC, and LysoPE, upregulates PPAR-γ expression, and inhibits the TLR-4/NF-κB signaling pathway	SD rats	2 g/kg	*In vitro*	Li et al. (2026)
Anti-insomnia	Increases the levels of neurotransmitters 5-HT and DA, decreases the levels of NE and Glu and the Glu/GABA ratio, and increases the level of GABA.	C57BL/6J mice	1000, 2000, 3000 mg/kg	*In vivo*	Tang et al. (2023)
	Improvement of tryptophan and GP metabolism and regulation of the gut microbiome	ICR mice	50, 100 mg/kg	*In vivo*	Yan et al. (2025)
	Restoring intracellular ATP and Ca^2+^ homeostasis to regulate upstream AMPK signaling	CTN-TNA2 cells	1 mg/mL	*In vitro*	Ma J et al. (2025)
	Increase GABA levels, decrease Glu levels, and improve the GABA/Glu balance	SD rats	4.05, 8.1 g/kg	*In vivo*	Bian et al. (2025)
	Increasing GABA levels improves sleep quality	BALB/c mice	0.57 g/kg	*In vitro*	Ren et al. (2023)
	Upregulating the expression of GABAA receptor α1 and γ2 subunits in the hypothalamic GABAergic system	HT22 cells; SD rats	4.5, 9.01, 13.5 mg/kg	*In vitro* and *vivo*	Xiao et al. (2022)
​	Increases the content of 5-HT and enhances the expression of AR and PPARG proteins in the brain	SD rats	156.25, 781.25, 1562.5 mg/kg	*In vivo*	Pan et al. (2025)
	Regulating the PI3K/AKT pathway, upregulating GABA BMAL1 and CLOCK and 5-HT levels, mRNA expression, and p-PI3K and p-AKT protein expression	BALB/c mouse; 4T1 mouse breast cancer cell line	2.5 g/kg	*In vivo*	Gu et al. (2026)
	Inhibition of the TLR4/NF-κB/NLRP3 pathway the regulates expression of hypothalamic neurotransmitters and inflammatory mediators	BALB/c mice	15.34, 30.68 g/kg	*In vivo*	Ma Z et al. (2025)
	Inhibition the p38MAPK/NF-κB signaling pathway	SD rats	3.0 g/kg	*In vivo*	Ji et al. (2024)
	Balance the ratio of Glu and GABA, regulate the synthesis and release of 5-HT, and modulate the expression level of the neurotransmitter NE.	Mice, Inbred ICR	20, 40, 80 g/kg	*In vivo*	Zhu Y et al. (2024)
	Treat insomnia by down-regulating the expression of TXNIP/NLRP3 proteins and regulating oxidative stress levels	SD rats	1.5, 3, 6 g/kg	*In vivo*	Xu et al. (2024)
	Adjusting the PI3K/AKT/BDNF signal path	SD rats	3.25, 7.50, 15 g/kg	*In vivo*	Deng et al. (2024)
	Activation of cAMP/CREB/BDNF and PI3K/Akt signaling pathways	SD rats	10, 20 g/kg	*In vivo*	Yuan et al. (2024)
	Adjust HPA axis function	SD rats	5, 20 g/kg	*In vivo*	Hua et al. (2022)
	Hypnotic may be related to the activity serotonergic system	ICR mice; SD rats	9 mg/kg	*In vivo*	Cao et al. (2010)
Anti-anxiety	Reduce NMDAR and AMPAR expression levels and improve synaptic plasticity	SD rats	10 g/kg	*In vivo*	Wang et al. (2023)
Neuroprotective activity	Upregulate the PI3K/Akt/mTOR signaling pathway	SD rats	3.6, 7.2, 14.4 g/kg	*In vivo*	Yi et al. (2025)
Vascular protection	Regulating the VEGFR2-PI3K/AKT/NF-Κb signaling pathway, inhibit inflammatory responses	hCMEC/D3 cells	0.2 g/mL	*In vitro*	Li et al. (2025)
​	Activation of the PI3K/Akt signaling pathwayregulates Bax/Bcl-2/caspase-3-mediated apoptosis	APP/PS1 transgenic mice; C57BL/6J mice	12.96,25.92 g/kg	*In vivo*	Wu et al. (2026)
Improve cognitive function	Activation of the PI3K/Akt signaling pathway reduces hippocampal neuronal apoptosis levels	SD rats	20 mg/kg	*In vivo*	Huang Z et al. (2025)
​	Improves learning and memory performance in rats, increases levels of 5-HT, GABA, and NO, decreases serum levels of IL-6, TNF-α, and Glu, and increases levels of IL-1β and MT.	SD rats	10, 20 g/kg	*In vivo*	Niu et al. (2025)
Other pharmacological activities	Increased miR-34a-5p expression, decreased 5-HT2AR expression, and enhanced 5-HT and 5-HT1AR binding activity	KM mouse	0.32 mg/g	*In vivo*	Pei et al. (2025)
	Induce endogenous apoptosis signaling pathways to activate apoptosis, inhibit the invasion of prostate cancer cells by affecting the expression of proteins such as snail, N-cadherin, and E-cadherin	DU145, VCaP cells	0.5.1 μmol/L	*In vitro*	Wang et al. (2025)
	Regulates gut microbiota composition and expression of autophagy-related genes in the liver and kidneys, and enhances the activity of liver and kidney antioxidant enzymes	KM mouse	100, 400 mg/kg	*In vivo*	Cao and Liu (2025)
	Inhibiting the NF-κB pathway	C57BL/6 mice	40 mg/kg	*In vivo*	Zhang and Zhang (2024)
	Adjusting miR-223-3p/SGK1/NLRP3 axes	293T, HK-2 cells; C57BL/6J mice	20, 40 mg/kg	*In vitro* and *vivo*	Huang et al. (2024)
	Inhibiting CD38 expression improves energy metabolism disorders and mitochondrial function, and alleviates inflammatory responses	H9C2 cells	40 μmol/L	*In vivo*	Hu et al. (2024)
	Activation of the AMPK/SIRT1/PGC-1 Signaling Pathway	APP/PS1 mice C57BL/6JNju mice	12.96, 25.92 g/kg	*In vitro*	Long (2023)
	Activation of the AMPK/SIRT1/PGC-1 Signaling Pathway	SD rats; H9c2 rat cardiomyocytes	0.675, 1.35, 2.7 g/kg	*In vitro* and *vivo*	Bi et al. (2025)

## 2 Methodology

Keywords, including *Ziziphi Spinosae Semen, Suanzaoren, jujube*, depression, anxiety depression, severe depression, adolescent depression, etc., were used to retrieve relevant literature and data through digital resources and print materials. The earliest literature was published in 1961, and the most recent in 2026. Existing literature has been systematically reviewed and discussed on the material basis and main bioactive metabolites of ZSS in the intervention of depression, and its potential mechanism of action has been further analyzed. Most literature searches were conducted through the following online scientific databases: PubMed, Web of Science, Google Scholar, CNKI, Baidu Scholar, China Science and Technology Journal Database, Wanfang Data Knowledge Service Platform, etc.

## 3 Ethnopharmacology and traditional uses

**FIGURE 2:**
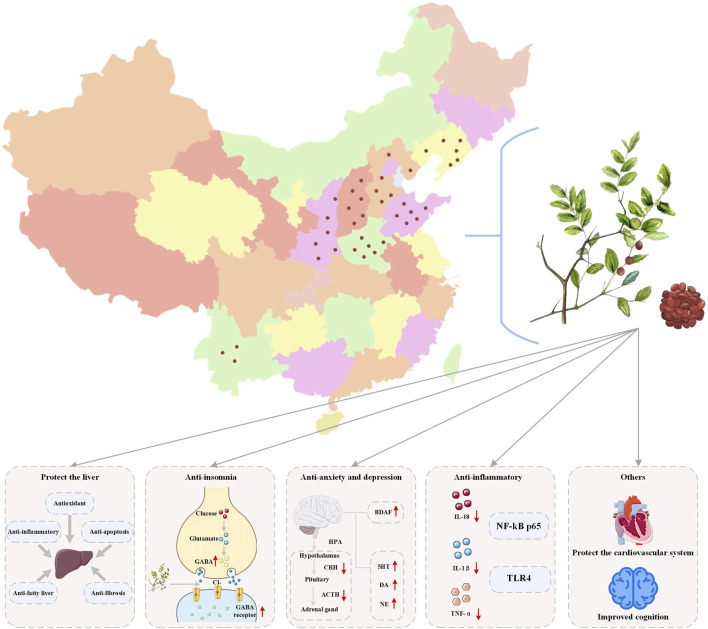
Origin and function of ZSS.

ZSS, also known as *sour jujube kernel, jujube kernel, thorn kernel*, and *jujube seed*, is extensively scattered throughout northern China, including Hebei, Shanxi, Shandong, Henan, and Liaoning. Dried, mature jujube kernels have medicinal and edible properties, making them one of the first Chinese medicinal botanical drugs publicly listed by the Ministry of Health as both medicinal and edible. They are also a representative traditional Chinese sedative and tranquilizing botanical drug (Liu M.-H. et al., 2024; Zhu et al., 2023). The seeds of Ziziphus jujuba possess sweet, sour, and neutral characteristics, and they influence the liver, gallbladder, and heart meridians. They invigorate the heart and liver, soothe the psyche, and facilitate perspiration and fluid generation. They are primarily used to treat symptoms such as insomnia, restlessness, spontaneous sweating, night sweats, and thirst due to fluid loss (Figure 2). Its medicinal value was first recorded in the Shennong Bencao Jing (Shennong’s Classic of Materia Medica), where it was rated as a top-grade medicinal botanical drug. It “treats restlessness and insomnia, excessive sweating due to heart deficiency, tonifies the middle, replenishes Qi, strengthens the bones and muscles, and supports Yin Qi. Long-term use can lighten the body and prolong life.” The Compendium of Materia Medica further states: “ZSS is sweet and moist, so cooked, it treats symptoms of gallbladder deficiency, insomnia, thirst, and sweating; raw, it treats gallbladder heat and promotes sleep”. Both are medicinal botanical drugs for the Jueyin and Shaoyang meridians of the foot. Modern people primarily consider them to be heart-related, ignoring this principle.” This statement clearly indicates that ZSS is closely linked to the regulation of the liver and gallbladder meridians, suggesting that its preparation method and dosage form should be appropriately selected based on the specific syndrome. Furthermore, the Leigong Paozhi Lun (Thoughts on Processing and Treatment) states: “When using ZSS, after harvesting, sun-dry them. Take the leaves, mix them with ZSS, steam them for half a day, remove the tips and peels, and grind them for use.” This detailed discussion of the medicinal properties of ZSS.

Modern pharmacological research has revealed that ZSS is abundant in diverse active metabolites, including saponins, flavonoids, alkaloids, and triterpenoids (Yan et al., 2019). These metabolites exhibit sedative, hypnotic, antidepressant, anxiolytic, and anticonvulsant effects, demonstrating significant potential for application in food, healthcare, and clinical medicine (Wang et al., 2022; Song et al., 2023; Hua et al., 2021). Currently, the Chinese Pharmacopoeia includes over 30 ZSS prescriptions, which are widely used in the clinical treatment of conditions such as palpitations, insomnia, anxiety, depression, and dizziness (Bian et al., 2021; Xiao et al., 2022). Subsequent investigations have shown that ZSS can exert therapeutic effects on neurological disorders through multiple mechanisms, including regulating neurotransmitter metabolism, improving synaptic plasticity, and modulating HPA axis function. The synergistic effects of these mechanisms, particularly in improving mood disorders, provide an important theoretical basis and potential application for the use of ZSS in the prevention and treatment of depression (Zhang et al., 2022; Du C. et al., 2024). Its widespread distribution and edible nature facilitate its clinical promotion and application. Furthermore, its multi-metabolite, multi-target, and multi-pathway pharmacological properties demonstrate the unique advantages of TCM in its holistic approach to regulation. More importantly, its good safety and tolerability, coupled with its characteristics of being both medicine and food, provide theoretical support for long-term use or dietary intervention, and also provide direction for the development of antidepressant products with dual healthcare and therapeutic functions.

## 4 Antidepressant bioactive metabolites of ZSS

**FIGURE 3:**
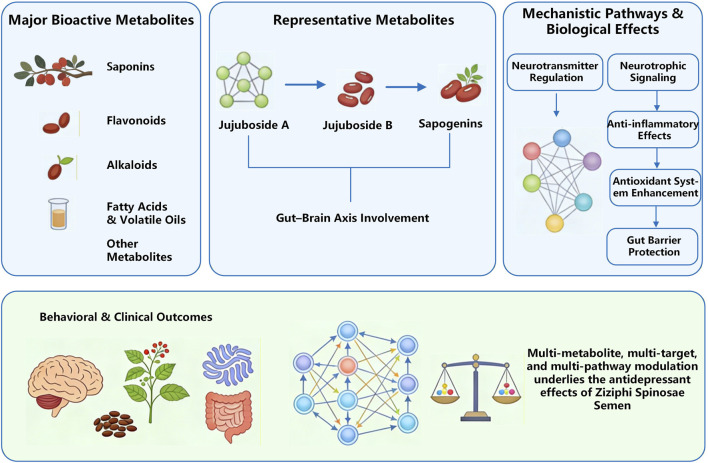
The main active metabolites in ZSS.

As a traditional Chinese medicinal material, the chemical composition of ZSS has a long history of research. To date, more than 150 chemicals have been extracted and characterized from the seeds, predominantly comprising triterpenoid saponins, flavonoids, alkaloids, fatty acids, and volatile oils (Liu et al., 2018) (Figure 3; Table 2). Triterpenoid saponins, flavonoids, alkaloids, and fatty acids have demonstrated significant pharmacological potential in neurological disease research, particularly regarding their anti-anxiety and anti-depressant effects, garnering considerable interest from scholars globally.Existing clinical research data supports, to some extent, the effect of ZSS formulas on improving depressive symptoms. Studies have shown that ZSS extract can improve sleep quality and mood in patients with insomnia or mild to moderate depression, preliminarily confirming the antidepressant potential of saponins and flavonoids. Meanwhile, some individual reports on the mood-improving and anxiety-reducing effects of ZSS extract taken according to traditional Chinese medicine methods provide additional clinical evidence (Dong et al., 2025). Studies by Zhang Guangping et al. have confirmed that venlafaxine combined with modified ZSS decoction for treating patients with depression and insomnia can further alleviate depressive mood on the basis of conventional antidepressant treatment, significantly improve sleep quality and quality of life, and no obvious safety issues were observed, suggesting that ZSS formulas have synergistic potential (Zhang G et al., 2025). By combining these data, we can conduct a more comprehensive assessment of the clinical application value of ZSS and its active ingredients, and lay the foundation for future high-quality interventional studies.

**TABLE 2:** The main chemical metabolites of ZSS.

Metabolites	Chemical formula	Molecular weight	References
Jujuboside A	C58H94O26	1207.3	Otsuka et al. (1978)
Jujuboside B	C52H84O21	1045.2	Otsuka et al. (1978)
Jujuboside A1	C58H94O26	1207.3	Masayuki et al. (1997)
Jujuboside B1	C52H84O21	1045.2	Masayuki et al. (1997)
Jujuboside C	C59H96O27	1237.4	Masayuki et al. (1997)
Acetyljujuboside B	C54H86O22	1087.2	Masayuki et al. (1997)
Protojujuboside A	C64H10O32	1387.5	Masayuki et al. (1997)
Protojujuboside B	C58H96O27	1225.4	Masayuki et al. (1997)
Protojujuboside B1	C58H96O27	1225.4	Masayuki et al. (1997)
Jujuboside D	C58H96O26	1207.3	Liu and Wang (2004)
Spinosin	C28H32O15	608.5	Woo et al. (1979a)
6‴-Feruloylspinosin	C38H40O18	784.7	Ding et al. (2011)
Nicotiflorin	C27H30O15	594.5	Ding et al. (2011)
Genkwanin	C16H12O5	284.26	Ding et al. (2011)
Apigenin	C15H10O5	270.24	Ding et al. (2011)
Isovitexin	C21H20O10	432.4	Ding et al. (2011)
Swertisin	C22H22O10	446.4	Woo et al. (1979a)
Lysicamine	C18H13NO3	291.3	Sun et al. (2013)
Juzirine	C17H15NO3	281.3	Sun et al. (2013)
Frangufoline	C31H42N4O4	534.7	Sun et al. (2013)
Nuciferine	C19H21NO2	295.4	Sun et al. (2013)
Coclaurine	C17H19NO3	285.34	Sun et al. (2013)
Palmitic acid	C16H32NO2	256.42	Liu J et al. (2015)
Arachidonic acid	C20H32O2	304.5	Liu J et al. (2015)
Myristic acid	C14H28O2	228.37	Liu J et al. (2015)
Pentadecanoic acid	C15H30O2	242.4	Liu J et al. (2015)
Palmitoleic acid	C16H30O2	254.41	Liu J et al. (2015)
Heptadecanoic acid	C17H34O2	270.5	Liu J et al. (2015)
Stearic acid	C18H36O2	284.5	Liu J et al. (2015)
Oleic acid	C18H34O2	282.5	Liu J et al. (2015)
Lauric acid	C12H24O2	200.32	Liu J et al. (2015)
Docosanoic acid	C22H44O2	340.6	Liu J et al. (2015)

### 4.1 Saponins

JuA, B, and C were initially extracted from the methanolic extract of ZSS 47 years ago. These metabolites are structurally highly similar to active saponins found in Panax notoginseng and Panax ginseng. Saponins are the primary metabolites of ZSS, whose sapogenins are categorized as tetracyclic and pentacyclic triterpenes. Currently, over 30 saponin metabolites have been identified in this botanical drug. Studies have shown that jujubosides exert potent antidepressant effects in numerous experimental studies through multiple targets and pathways. JuA and JuB are the most representative. JuA has exhibited robust antidepressant effects in animal models, markedly reducing immobility duration in forced swim and tail suspension assays in depressed mice. It also exerts anti-anxiety and anti-depressant effects by increasing expression of BDNF, its receptor TrkB, and CREB in the mouse hippocampus (Li H. et al., 2022). Subsequent research has demonstrated that JuA can activate the Shh signaling pathway via modulating calcium homeostasis and synaptic plasticity in immature neurons, consequently ameliorating depressive-like behavior and cognitive impairment in rats subjected to the CUMS paradigm (Zhong et al., 2024). In addition, JuA also has good sedative and hypnotic effects, and affects the cytokine network between brain nerve cells by regulating the expression of GABA receptor subunit mRNA and downregulating the secretion of related inflammatory cytokines in the intestinal mucosal system (Wang et al., 2015). In contrast, JuB has attracted widespread attention from scholars at home and abroad as an alternative chemical preventive and therapeutic drug. Within the established safe dosage parameters, JuB demonstrates substantial clinical acceptability and greatly mitigates depressive-like symptoms in mice by elevating levels of 5-HT and Trp, while effectively reversing tumor development generated by CUMS (Yang et al., 2022). Further studies have confirmed that JuB is one of the main metabolites of JuA in the intestine. JuA is first hydrolyzed into JuB, which can then be further metabolized into jujube saponin by the intestinal microbiota of rats, thereby exerting its biological effects. In addition to JuA and JuB, other saponin metabolites also shown good antidepressant potential, indicating that saponins are an important material basis for the central nervous system regulation effect of ZSS.

From a structural pharmacology perspective, the main antidepressant active metabolites in ZSS share structural and functional similarities with commonly used clinical antidepressants in terms of molecular skeleton, functional group distribution, and lipid solubility. This provides an important chemical basis for their neuropsychopharmacological activity. JuA and JuB belong to the triterpenoid saponin class, with a core pentacyclic triterpenoid skeleton exhibiting a distinct steroid-like stereostructure. These molecules typically consist of a hydrophobic saponin aglycone and a hydrophilic sugar chain, displaying amphiphilic characteristics. This structure facilitates their interaction with the cell membrane lipid bilayer, regulating membrane receptor conformation and signal transduction pathways. Commonly used anti-anxiety drugs, such as Diazepam, exert their effects by enhancing GABAA receptor activity (Reynolds et al., 2001). Although JuA differs significantly from benzodiazepines in skeletal structure, studies have shown that JuA can regulate GABAA receptor subunit mRNA expression, and its indirect regulatory mechanism shows functional convergence with the “positive allosteric regulation” of benzodiazepines. Furthermore, the spatial conformation of the triterpenoid skeleton shares structural similarities with some neurosteroids, which have been shown to regulate GABAA receptor and HPA axis function. Therefore, saponins may participate in mood regulation through a neurosteroid-like mechanism.

### 4.2 Flavonoids

Flavonoids are one of the main active metabolites in the ZSS, with a total flavonoid mass fraction of approximately 0.95%. They are a class of polyphenolic metabolites widely found in nature. As early as the late 1970s, Woo et al. (1979b) first isolated the spinosin flavonoid carbonyl glycoside from the seeds of the ZSS and determined its structure. Subsequently, numerous flavonoid metabolites have been isolated from the seeds. To date, 44 flavonoid-related metabolites have been identified in the seeds of the ZSS, including 31 flavonoids, eight flavonols, two dihydroflavonoids, and three isoflavones. Most of these flavonoids exist as glycosides, primarily carbonyl and oxygen glycosides, with a small amount of nitrogen glycosides also being detected (Wen et al., 2021; Li et al., 2016). Pharmacological research has demonstrated that flavonoids derived from ZSS exhibit a variety of biological activities in the central nervous system, including sedative and hypnotic effects, antidepressant and anxiolytic effects, antioxidant properties, and learning and memory enhancement. They can significantly improve depressive-like behaviors in animal models (Liu Y et al., 2015; Zhang J et al., 2020). Mechanistic investigations have demonstrated that flavonoids improve intestinal barrier function by increasing levels of 5-HT and its precursor 5-HTP in the hippocampus, while simultaneously downregulating the expression of ZO-1, Claudin-1, and Occludin, along with TNF-α, IL-6, and IL-1β in the colons of mice exposed to chronic restraint stress. This, in turn, improves anxiety-and depression-like behaviors in mice and exerts a sedative and hypnotic effect (Yan et al., 2025). Among the numerous flavonoids, spinosin is considered the most representative core metabolite. Studies have demonstrated that spinosin plays a key role in alleviating chronic restraint stress-induced anxiety and depression by regulating the ERK1/2/CREB/BDNF signaling pathway, thereby reducing inflammation and neuronal damage in the hippocampus and prefrontal cortex of C57BL/6J mice (Guo et al., 2024). Furthermore, Li et al. (2016) reported that the typical flavonoid swertisin exhibits sedative effects similar to those of diazepam, achieving its pharmacological effects by inducing the expression of GABA receptor subunits.

Flavonoids (such as Spinosin) possess a typical C6–C3–C6 flavonoid core structure, containing a polyphenolic hydroxyl group and an aromatic ring system with strong *π* -electron conjugation (Wu et al., 2025). Structurally, this aromatic ring system shows some electronic similarity to the aromatic ring structures of SSRIs such as Fluoxetine and Sertraline. SSRIs typically contain hydrophobic aromatic groups and amino side chains for binding to 5-HT transporters. Although flavonoids lack typical amino structures, the hydrogen bond donor-receptor system formed by their aromatic rings and hydroxyl groups may affect monoamine metabolism-related proteins through weak interactions. Furthermore, flavonoids possess antioxidant and anti-inflammatory capabilities and can inhibit the NF-*κ*B signaling pathway, echoing the “inflammation hypothesis” proposed in recent years, while SSRIs have also been shown to have certain adjuvant anti-inflammatory effects. Therefore, there is mechanistic convergence between the two at the level of “neuro-inflammatory-monoamine system cross-regulation.”

### 4.3 Alkaloids

ZSS is not only rich in saponins and flavonoids, but also contain a significant amount of alkaloids, which have garnered considerable research attention in recent years. As early as 1997, Yin et al. first isolated two alkaloids from ZSS—lysicamine and juzirine—marking the first discovery of alkaloids from this plant (Yin S.Z et al., 1997). Researchers have effectively extracted and identified more than 20 alkaloids, predominantly cyclic peptides (including sanjoinine A, B, D, F, and G1) and isoquinolines (such as nuciferine, coclaurine, nornuciferine, and norisocorydine). Experimental studies have shown that ZSS alkaloids significantly reduce brain NO levels and enhance T-AOC, GSH-PX, and CAT activities in a CUMS mouse model of depression. Their antidepressant effects are mainly accomplished by inhibiting monoamine oxidase activity, scavenging free radicals, and preventing lipid peroxidation (Sun Y, 2014). Network pharmacology analysis further revealed that alkaloids from ZSS not only possess independent antidepressant activity but also synergize with other active metabolites (Xia M.L et al., 2023). Animal tests demonstrated that, in comparison to the control group, co-administration of alkaloids and saponins significantly shortened the immobility time of mice in behavioral tests and increased levels of NE, DA, and 5-HT in the hippocampus and prefrontal cortex, significantly ameliorating anxiety and depressive-like behaviors in murine models (Li L. et al., 2022). Moreover, research indicates that the coadministration of magnolidine and spinosin alters the oil-water partition coefficient, enhancing the lipid solubility of magnolidine while simultaneously augmenting the water solubility of spinosin, thereby facilitating their passage across the blood-brain barrier into the central nervous system. This synergistic impact markedly enhances cell survival and further extends immobility duration in the tail suspension test and forced swim test in CUMS mice (Bi et al., 2024). In summary, existing studies have fully demonstrated the multi-target pharmacological mechanisms and significant efficacy of ZSS alkaloids in antidepressant treatment, establishing a robust theoretical and empirical basis for the clinical advancement of safer and more efficacious antidepressant medications.

Alkaloids contain nitrogen-containing heterocyclic structures, a feature highly similar to that of most classic antidepressants. For example, the tricyclic antidepressants imipramine and amitriptyline both contain aromatic tricyclic structures and tertiary amine side chains, and their antidepressant effects are closely related to the inhibition of monoamine reuptake. Isoquinoline alkaloids also possess aromatic heterocycles and nitrogen atoms, which can act as weakly basic centers to participate in receptor binding or enzyme inhibition. Previous studies have suggested that ZSS alkaloids can inhibit monoamine oxidase (MAO) activity, thereby increasing the levels of 5-HT and NE in the brain. This mechanism is highly consistent with the pharmacological pathway of monoamine oxidase inhibitors (MAOIs).

### 4.4 Fatty acids and volatile oils

Jujube kernel oil is a lipid-rich substance derived from the ZSS. It comprises a diverse array of 105 chemical metabolites, including fatty acids, lipids, alcohols, and alkyl hydroxyls. Fatty acids are the primary metabolite of jujube kernel oil (Ma et al., 2021). As a TCM kernel, the fatty acid content of jujube kernels can reach 32%, encompassing 17 different types. Unsaturated fatty acids account for a whopping 80.2%, predominantly comprising arachidic acid, myristic acid, lauric acid, palmitic acid, stearic acid, linoleic acid, pentadecanoic acid, hexadecenoic acid, and docosanoic acid. Contemporary pharmacological studies have demonstrated that jujube kernel oil not only possesses antioxidant, lipid-lowering, anti-tumor, and cognitive-enhancing properties, but also demonstrates outstanding sedative, hypnotic, anti-anxiety, and anti-depressant properties. It is also safe for long-term use, with no significant toxicity or mutagenic risks (Jia Y et al., 2018). Further mechanistic studies have found that terpenoids isolated from Chinese jujube kernel oil can significantly increase serotonin levels, promote GABA synthesis and GABA A receptor expression, inhibit the overexpression of Glu and NE, and simultaneously downregulate IL-1β levels and upregulate the activity of SOD and inducible nitric oxide synthase, thereby regulating neurotransmitters and inflammatory responses from multiple targets, thereby improving mood (Sun et al., 2024). Oleamide may mitigate oxidative stress responses and modulate energy metabolism, resulting in substantial improvements in both acute stress models and chronic moderate stress depression models, consequently exhibiting pronounced antidepressant efficacy (Ge et al., 2015). As a significant active metabolite of the ZSS, Chinese jujube kernel oil has multiple pharmacological advantages in neurotransmitter regulation, anti-oxidation, and anti-inflammation, providing a solid experimental basis for its clinical application in mood disorders, especially depression, and also laying a good research foundation for the development of new Chinese medicine preparations.

### 4.5 Other metabolites

In addition to the main active metabolites described above, ZSS is abundant in polysaccharides, phytol, phenolic acid, vitamin C, and a significant quantity of cyclic adenosine monophosphate. ZSS contains seven vital trace elements: iron, manganese, copper, zinc, chromium, selenium, and nickel, along with eight essential amino acids, including tyrosine, methionine, valine, and threonine. They are crucial for sustaining metabolic equilibrium, modulating immunological function, and enhancing the health of the neurological system (Zhou et al., 2023). Systematic research on the chemical composition of ZSS not only helps to identify its active sites and identify key substances closely related to pharmacological effects, but also provides a material basis for elucidating its clinical application mechanism. It can be said that the comprehensiveness and accuracy of chemical composition analysis directly affects the depth of the revelation of pharmacological mechanisms and the scientific nature of clinical applications.

## 5 Antidepressant mechanism of action of ZSS

**FIGURE 4:**
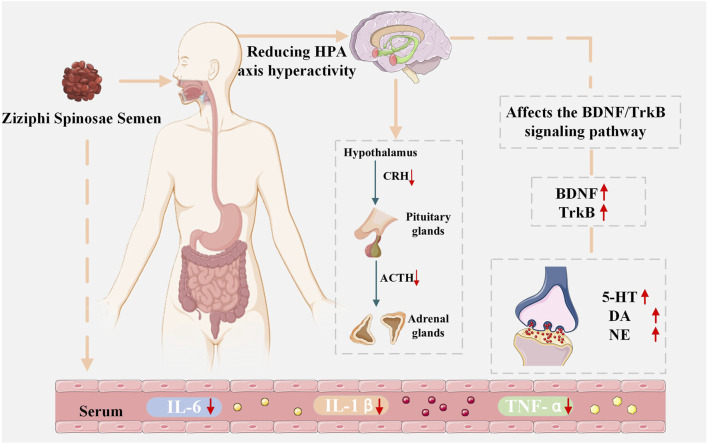
Diagram of the antidepressant mechanism of ZSS.

Depression falls under the categories of “depression,” “lily disease,” and “plum pit qi” in TCM. Depression in TCM refers to a disorder characterized by stagnation of Qi and the imbalance of Yin and Yang, Qi, and blood in the internal organs, caused by preexisting liver hyperactivity or constitutional weakness, manifested by emotional distress. Symptoms include depression, restlessness, irritability, and a sensation of a foreign body obstructing the throat. Depression often originates in the liver but can also affect the heart, spleen, lungs, and kidneys. The “Yi Fang Lun - Yueju Pills” states that “all depression must first be caused by Qi disorder. If Qi is flowing freely, there will be no depression,” emphasizing that regulating Qi is crucial for prevention and treatment. Contemporary pharmacological studies indicate that ZSS and its bioactive metabolites can have antidepressant effects by modulating the equilibrium of monoamine neurotransmitters in the central nervous system, modulating HPA axis function, promoting BDNF expression, and inhibiting inflammatory cytokines (Liu et al., 2012; Huang et al., 2018; Ma et al., 2017) (Figure 4).

### 5.1 ZSS regulates the homeostasis of monoamine neurotransmitters

Monoamine neurotransmitters are pivotal in sustaining homeostasis and modulating the formation and plasticity of brain circuits associated with mood disorders. Their metabolic imbalance is considered a key pathogenesis of depression. Numerous studies have established that variations in the levels of monoamine neurotransmitters, including DA,5-HT, and NE, are significantly linked to the onset of depression (Qian et al., 2022; Roubertoux et al., 2020; Chen, 2020; Bhatt et al., 2021; Shao and Zhu, 2020). Specifically, 5-HT is extensively distributed throughout the cerebral cortex and synaptic connections, participating in the regulation of volitional activity and emotional stress; NE is primarily released by the locus coeruleus in the brainstem, regulating learning and memory, sleep, and neuroendocrine function; and DA is secreted by the substantia nigra and plays a key role in the transmission of neural excitation signals. Both clinical and animal studies have found that the number of 5-HT, NE, and DA receptors in the synaptic cleft and postsynaptic membrane in the brains of patients with depression is significantly reduced, suggesting that regulating the homeostatic balance of monoamine neurotransmitters may be an important approach for antidepressant treatment. In pharmacological studies, a suspension of ZSS and lily powder was found to significantly increase peripheral blood serum 5-HT levels and brain 5-HIAA content, effectively alleviating depressive-like behaviors in depression models (Wang et al., 2017). Cheng et al. conducted a further examination of the effects of ZSS extract on a CUMS-induced model of depression and sleeplessness in murine subjects. They found that ZSS extract increased 5-HT and 5-HIAA levels in the serum and thalamus of mice to varying degrees, while significantly decreasing levels of CRH, ACTH, and CORT, and effectively modulated the expression of related proteins (Cheng H.B et al., 2025). The findings indicate that ZSS extract may produce its antidepressant effects via many pathways, including regulating 5-HT metabolism and levels of hormones related to the HPA axis, reducing hippocampal pathological damage, and improving synaptic plasticity. Furthermore, other experiments have shown that a combination of ZSS and *Polygala tenuifolia* exerts a sedative and hypnotic effect by regulating 5-HT, NE, and DA levels in total brain protein in mice (Luo et al., 2020). In summary, ZSS and its metabolite preparations have shown great potential in regulating the levels of monoamine neurotransmitters, providing a multi-target intervention pathway for the prevention and treatment of depression. This not only confirms the value of TCM in the treatment of mood disorders, but also provides an entry point for modern pharmacological mechanism research.

### 5.2 ZSS regulates HPA axis function

The HPA axis is a core neuroendocrine system that regulates homeostasis, regulates stress responses, participates in the regulation of glucose and lipid metabolism, and influences mood and cognitive function (Miller, 2018). When the body is exposed to external stimuli, the HPA axis is rapidly activated, with a significant increase in the secretion of CRH and GCs. Numerous studies have shown that persistent stress can lead to HPA axis hyperfunction, an abnormal state closely associated with depression and cognitive impairment. Clinical statistics show that approximately 40%–60% of patients with depression experience HPA axis dysfunction, primarily through mechanisms including persistently elevated cortisol secretion and decreased negative feedback regulation of GC receptors. Elevated cortisol levels are positively correlated with depression severity and are particularly pronounced in melancholic depression (Menke, 2024). Therefore, HPA axis hyperfunction has been recognized as a key link in the development and progression of depression. In clinical practice, ZSS is frequently combined with other herbal formulas to create effective prescriptions. The modified ZSS decoction is the most widely used of these. Further research has shown that modified ZSS Decoction can regulate the expression of orexin-A, maintaining HPA axis homeostasis and the balance of related neurotransmitters. This increases the levels of 5-HT and 5-HTR1A proteins in the hypothalamus, decreases DA and NE levels, and downregulates hormones such as CORT, ACTH, and CRH, as well as serum orexin-A concentrations (Dong et al., 2021). Furthermore, research by Wang et al. suggests that metabolite ZSS capsules (ZSS and levorotatory tetrahydropalmatine) can exert their effects by regulating metabolic disorders in neurotransmitters, the HPA axis, inflammatory factors, and the cAMP/CREB signaling pathway (Wang et al., 2024).

### 5.3 ZSS promotes BDNF expression

BDNF is an important neurotrophic factor involved in regulating cell growth, development, differentiation, and apoptosis. It is also a key signaling molecule that maintains normal development and function of the nervous system. BDNF is predominantly expressed in the central nervous system, as well as in the endocrine system, bone, and cartilage. Research indicates that reduced expression of BDNF and other neurotrophic factors may result in atrophy of critical tissues within the limbic system, such as the hippocampus and prefrontal cortex, thereby affecting neuronal proliferation, differentiation, and survival, and increasing the risk of depression (Li et al., 2021). Therefore, BDNF is widely considered a key biomarker of depression. Jiang et al. found that decreased BDNF expression leads to morphological and functional changes in hippocampal and cortical neurons, facilitating the advancement and evolution of depression (Jiang et al., 2014). Concerning TCM interventions, ZSS decoction has demonstrated the ability to enhance neuronal proliferation and produce antidepressant effects by upregulating the gene expression of BDNF and its receptor, TrKB (TIAN et al., 2014). Tomohiro Umeda et al. found that ZSS powder can increase BDNF expression, which is involved in repairing damaged neurons, and promote neurogenesis, improving cognitive function, preventing dementia, and promoting brain rejuvenation (Umeda et al., 2025). Shi et al. used a CUMS rat model of depression to investigate the antidepressant mechanism of a combination of ZSS and Albizia julibrissin. The results showed that compared with the control group, CUMS rats exhibited decreased cognitive performance, significantly reduced serum BDNF levels, and significantly downregulated hippocampal expressions of CREB mRNA, BDNF mRNA, and ERK, phosphorylated ERK, p-RSK, and p-CREB proteins. Treatment with ZSS and Albizia julibrissin significantly improved these markers, suggesting that their antidepressant effects may be achieved by enhancing the activity of key molecules in the BDNF/MEK/ERK/CREB signaling pathway (Shi et al., 2019). Another study demonstrated that an ethanol extract of ZSS reduced forced swimming immobility time, increased voluntary movement distance, and significantly increased hippocampal NE, 5-HT, and BDNF levels in CUMS mice, demonstrating a significant antidepressant effect (Oh et al., 2020). Furthermore, the ZSS and *Schisandra chinensis* herbal combination, a commonly used sedative and tranquilizing combination, has also demonstrated significant efficacy in animal models of depression. Related studies indicate that this medication combination can ameliorate anxiety-depression-like behaviors generated by restraint stress by modulating the expression of the ECS/BDNF/ERK signaling pathway (Liu et al., 2023). A comprehensive review of existing research indicates that ZSS and their combined medicinal metabolites can significantly increase the activity of BDNF and related signaling pathways in various animal models of depression, accompanied by improvements in neurotransmitter levels and behavioral indicators. This provides strong experimental evidence for the use of ZSS in the intervention of depression.

### 5.4 ZSS have anti-inflammatory effects

Inflammatory factors are important bioactive substances that mediate the body’s inflammatory response, primarily including interleukins and tumor necrosis factor. Proinflammatory mediators, including IL- 1*β*, IL-6, IL-8, and TNF-α, facilitate inflammatory responses, whereas anti-inflammatory mediators such as IL-4 and IL-10 suppress inflammatory processes. Multiple studies indicate that individuals with depression frequently exhibit compromised immune function and modified immune cell counts, suggesting a crucial role for the immune system in the development and progression of depression (Kofod et al., 2022; Chu et al., 2021). Chronic stress can persistently activate inflammatory responses. Sustained elevation of proinflammatory factors not only damages nerve cells and neurons but also alters brain structure and neuroendocrine function, thereby inducing depressive-like behaviors. In recent years, inflammatory factors have been recognized as important biomarkers of depression, and the assembly and activation of inflammasomes have been shown to be closely associated with the course of the disease. Animal experiments have shown that ZSS decoction can significantly reduce the expression of related inflammatory factors, alleviate histopathological damage in the hippocampus and colon, and correct intestinal microbiota imbalance in mice with depression, thereby improving depressive-like behaviors (Du et al., 2023). Using a CUMS-induced depression model in SD rats and a LPS-induced neuroinflammation model in BV2 cells, Du et al. further validated the antidepressant mechanism of ZSS decoction: it not only reduces inflammatory cytokine levels but also upregulates the expression of BDNF, SYP, and PSD95, and inhibits overactivation of the TLR4/MyD88/NF-*κ*B and Wnt/*β*-catenin signaling pathways, thereby alleviating anxiety-depressive-like behaviors (Du et al., 2024c). Another study has shown that ZSS extract can downregulate the levels of NLRP3, GSDMD, ASC, caspase-3, and caspase-8 proteins in the thalamus of CUMS mice, while upregulating the expression of HO1 and NRF2 proteins, thereby alleviating inflammation and oxidative stress (Cheng et al., 2025). ZSS and its preparations show great potential in inhibiting neuroinflammation, improving neuroplasticity and maintaining neural homeostasis through multi-pathway and multi-target regulatory effects.

## 6 Modern applications of ZSS

### 6.1 Antidepressant effects of ZSS in TCM

ZSS is a commonly used tranquilizer in TCM. With a neutral nature and sweet and sour flavor, it boasts benefits such as nourishing the heart and liver, calming the mind and relieving restlessness, and promoting sweating and salivation. Its medicinal value is widely documented in TCM formulas. Formulas using ZSS as a core metabolite are particularly common in the treatment of emotional disorders. Representative recipes include ZSS Decoction, Guipi Decoction, and Jieyu Anshen Decoction.

The decoction of ZSS was first referenced in Zhang Zhongjing’s Synopsis of the Golden Chamber during the Eastern Han Dynasty. It states, ZSS Decoction is the main treatment for symptoms of asthenia, restlessness, and insomnia.” It consists of five botanical drugs: ZSS, *Chuanxiong*, *Poria*, *Anemarrhena*, and *Licorice*. ZSS is the main botanical drug in this formula, nourishing blood and calming the mind; *Chuanxiong* activates blood circulation and promotes qi; *Poria* calms the heart and strengthens the spleen; anemarrhena clears heat and nourishes yin; and *Licorice* harmonizes the other botanical drugs. This mixture soothes the mind and is appropriate for conditions such as depression and sleeplessness (Zhang et al., 2021; Du et al., 2024b; Yan et al., 2023). Luo H.B et al. (2022) examined the clinical efficacy of Ganmai Dazao Decoction in conjunction with modified ZSS Decoction for treating depression associated with hepatic depression and spleen deficiency. A control group received fluoxetine hydrochloride, while an observation group received Ganmai Dazao Decoction combined with modified ZSS Decoction. The HAMD score in the observation group was significantly lower after treatment than in the control group (P *<* 0.05). Further clinical evidence suggests that modified ZSS Decoction is also suitable for the intervention of post-stroke depression in middle-aged and elderly women. It not only effectively improves depressive symptoms but also enhances quality of life and cognitive function. It is safe, effective, and shortens the treatment course (Dong et al., 2025). Liu L et al. (2024) randomly divided 60 adolescent patients with depression into a control group and a study group of 30 patients. Both groups received sertraline tablets, while the study group received ZSS Decoction in addition. Post-treatment, the HAMD-17 scores in the experimental group were markedly lower than those in the control group. The patients’ depression and anxiety were effectively alleviated, their sleep quality improved, and their social functioning improved significantly. No significant adverse reactions were observed, demonstrating high safety and clinical value. Furthermore, studies have reported that modified ZSS Decoction combined with auricular acupressure can effectively relieve anxiety and depression in patients with malignant tumors, while also alleviating cancer pain and improving psychological wellbeing and quality of life. This finding provides new insights and references for the comprehensive treatment of advanced cancers (Peng et al., 2021).

Guipi Decoction, originating from the Song Dynasty book “Jisheng Fang” by Yan Yonghe, uses ZSS combined with various botanical drugs to nourish qi and blood, calm the mind, and strengthen the spleen. It is frequently utilized to address sadness, sleeplessness, and amnesia resulting from deficiencies of both the heart and spleen. Its therapeutic approach focuses on regulating the production of qi and blood in the spleen and stomach, while also providing both tranquilizing and nourishing effects (Liu M. et al., 2024). An increasing array of research has shown that Guipi Decoction combination therapy is significantly effective in treating depression associated with various diseases. Its application encompasses clinical conditions such as breast cancer-related depression, post-stroke depression, elderly depression, and anxiety and depression associated with coronary heart disease. It not only effectively alleviates depressive symptoms but also offers advantages such as minimal side effects and a high safety profile, demonstrating broad research and application prospects. A study by Li M et al. (2025) demonstrated that Guipi Decoction combined with escitalopram significantly improved depression, anxiety, and sleep in patients with mild to moderate depression, with overall efficacy superior to escitalopram alone and a relatively favorable safety profile. Zhu et al. (2024) performed a randomized controlled experiment assessing the efficacy of Guipi Decoction in 98 individuals diagnosed with postpartum depression. The results showed that adding Guipi Decoction to conventional sertraline treatment not only improved overall treatment efficacy but also improved patients’ sex hormone levels and reduced TCM syndrome scores and depression scores. Furthermore, Jiao et al. (2023) conducted a clinical study on elderly patients with hypertension and depression associated with Qi and blood deficiency. The results showed that combined treatment with modified Guipi Decoction significantly alleviated depressive symptoms, while also lowering blood pressure and Hcy levels, with no significant adverse reactions, further validating its safety and clinical value. Notably, the combination of kidney-warming and governor-unblocking acupuncture with Guipi Decoction has been shown to play a positive role in improving sleep quality, regulating negative emotions, and alleviating depressive symptoms, thus providing new insights into multidimensional interventions for depression (Cui B.B, 2025).

Jieyu Anshen Decoction is a modified version of Xiaoyao San from the Taiping Huimin Hejijufang (prescriptions for the treatment of depression). Its metabolites include stir-fried ZSS, *Bupleurum chinense, Angelica sinensis, Polygonum multiflorum, White Peony Root, Toosendan Fruit, Atractylodes macrocephala, Curcuma aromatica, Poria cocos*, and *Licorice root*. ZSS calms the mind, *B. chinense* soothes the liver and relieves depression, *A. sinensis* nourishes and activates blood circulation, and *C. aromatica* promotes qi circulation and relieves depression. Combined with botanical drugs that strengthen the spleen and nourish the heart, the decoction is highly effective in relieving symptoms such as emotional distress, chest and flank distension, restlessness, and insomnia. Modern pharmacological and clinical studies have further confirmed that Jieyu Anshen Decoction combined with paroxetine is effective in treating depression. It not only significantly increases serum BDNF levels but also reduces inflammatory responses, thereby alleviating depressive symptoms. It also exhibits a favorable safety profile (Ke and Yang, 2025). Jiang et al. (2024) also demonstrated that this decoction is clinically effective as an adjunctive treatment for patients with Parkinson’s disease, sleep disorders, and depression. It effectively improves TCM syndromes and sleep quality, alleviates depressive symptoms, and regulates cytokine levels and monoamine neurotransmitter metabolism. More and more clinical studies have shown that the combined use of Jieyu Anshen Decoction with multiple intervention methods (such as scalp acupuncture, repetitive transcranial magnetic stimulation, psychological intervention and combined Western medicine treatment, etc.) can significantly enhance the therapeutic effect. Compared with a single treatment method, the combined treatment plan can not only more effectively relieve depressive symptoms, but also play a greater advantage in improving overall clinical symptoms and improving patients’ quality of life, showing broad clinical application prospects.

ZSS and related prescriptions hold a prominent place in TCM for the treatment of emotional disorders. Their modes of action predominantly involve nourishing the heart, calming the mind, soothing the liver, alleviating depression, and replenishing qi and blood. ZSS can be used alone as a main botanical drug to emphasize their calming and stabilizing effects, or combined with other botanical drugs to achieve multi-target, multi-step synergistic effects. In recent years, while inheriting the holistic approach of traditional prescriptions, modern medicine has combined pharmacological and clinical research to uncover the active metabolites and mechanisms of action of ZSS, providing a scientific basis for the modification and subtraction of prescriptions. This process has not only deepened our understanding of the mechanisms of ZSS in treating depression and other emotional disorders, but has also promoted their modern and standardized clinical application.

### 6.2 Application of ZSS in food and healthcare

**FIGURE 5:**
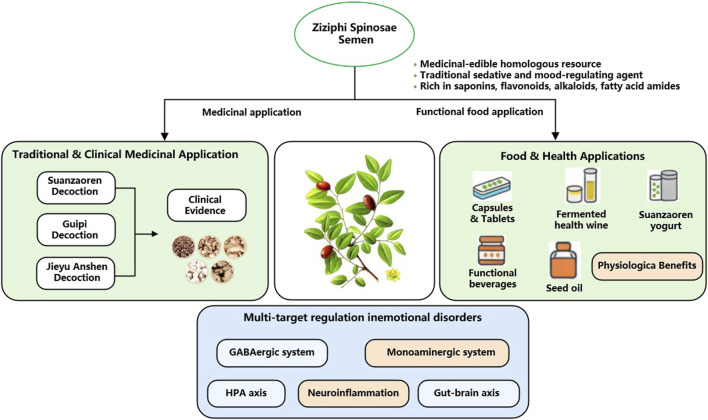
Application of Chinese medicinal material ZSS, which has the same origin as food and medicine, in related fields.

The ongoing comprehensive investigation into the chemical metabolites and pharmacological properties of ZSS, coupled with rising health awareness and growing acceptance of functional foods, is leading to the constant emergence of new ZSS-related health supplements and functional foods (Lin, 2021; Zhang S. et al., 2020) (Figure 5). These products are often added to herbal beverages, nutritional supplements, and specific functional foods, and are formulated in a variety of dosage forms, including capsules, oral liquids, chewable tablets, granules, and alcoholic solutions. Their combination of medicinal value and convenience has made them popular with consumers (Zeng et al., 2025). To meet modern consumer needs, ZSS has been formulated into more convenient and standardized products. For example, concentrated ZSS extracts can be packaged into capsules, providing accurate dosage and ease of use, avoiding the bitter taste of direct consumption. Alternatively, ZSS powder or extracts can be mixed with excipients and pressed into tablets, similar to standard tablets, making them easy to carry and prescribe. Modern fermentation technology has enabled the development of ZSS anti-nervous health wines. Microbial fermentation boosts the concentration and bioavailability of active metabolites, while also improving their nutritional value and pharmacological effectiveness (Liu D et al., 2020). Another typical product is jujube kernel yogurt. Made from jujube kernel homogenate or powder, it is fermented under various conditions with lactic acid bacteria, thermophilic *Streptococcus*, and other bacteria. It shares the health benefits of conventional dairy products, including maintaining intestinal flora balance and boosting immunity, while also retaining the antioxidant and free radical scavenging properties of jujube kernels and their unique flavor (Yun et al., 2011; Lin et al., 2020). Thanks to the rich flavonoids in jujube kernels and their calming and soothing properties, the development of functional beverages with depression-relieving and calming properties has become a key focus in the industry. These beverages, often made with fresh jujube juice, jujube kernel extract, and jujube leaf decoctions as primary metabolites, are formulated into liquid functional foods, satisfying modern consumers’ demand for natural, plant-based functional beverages (Feng Y et al., 2022; Xu Y et al., 2017; Liu et al., 2021; Li X et al., 2020). Furthermore, vegetable oil extracted from jujube kernels also holds significant application value. Jujube kernel oil, obtained through processes such as pressing, solvent extraction, or microwave/ultrasound-assisted extraction, is widely used in health foods, health supplements, cosmetics, and feed additives due to its nutrient-rich properties and pharmacological activity (Peng et al., 2017). The transition of ZSS from traditional Chinese medicinal materials to the modern food and healthcare field is a perfect embodiment of its value of “medicine and food having the same origin”. With its definite calming, tranquilizing, and anti-anxiety and depression effects, it is integrated into various forms such as porridge, tea, paste prescriptions, and capsules, providing modern people who are troubled by anxiety and depression with a natural and gentle conditioning option.

## 7 Conclusion and perspectives

In summary, ZSS, as one of the earliest medicinal and edible plants announced by the Ministry of Health, has been widely researched and applied in the fields of medicine, health food, cosmetics, and feed additives, demonstrating high application value and industrial prospects. In terms of chemical composition, triterpenoid saponins, flavonoids, various alkaloids, kernel oil rich in unsaturated fatty acids, polysaccharides, and trace elements collectively constitute the complex and synergistic material basis of ZSS, exhibiting multi-component, multi-target, and multi-pathway advantages in antidepressant and neuropsychiatric intervention. Pharmacological evidence shows that these components can regulate monoamine neurotransmitter homeostasis, inhibit and reverse HPA axis hyperfunction, upregulate BDNF/TrkB/CREB-related neurotrophic pathways, inhibit TLR4/NFB/NLRP3-mediated neuroinflammatory responses, and improve oxidative stress, thereby exerting specific functional effects of antidepressant, mood-improving, and sleep-promoting effects in animal models. Furthermore, the synergistic effects among natural components (such as the combination of saponins with flavonoids and alkaloids) and the metabolic transformation mediated by gut microbiota suggest that their *in vivo* effects depend not only on the chemistry itself, but also on the regulation of host metabolism and the microbial community.

This study systematically integrated traditional literature records, modern pharmacological experiments, and preliminary clinical research results to comprehensively analyze the potential mechanism of action of ZSS in the intervention of depression. Overall, existing evidence supports the pharmacological characteristics of ZSS, including multi-component synergy, multi-target regulation, and multi-pathway integration, and its direction of action is highly consistent with the current mainstream pathological hypothesis of depression. However, from the perspective of evidence-based medicine and translational medicine, related studies still suffer from insufficient levels of evidence and inadequate depth of mechanism verification. First, existing mechanism studies mostly focus on the correlation observation of changes in expression levels, with insufficient causal verification. For example, whether BDNF upregulation is a necessary condition for its antidepressant effect lacks verification through pathway blocking or gene knockout models. Similarly, the regulatory sequence and key nodes between inflammatory and neurotrophic pathways remain unclear. Therefore, future research should strengthen functional blockade experiments and multi-omics integrated analysis to construct a clearer mechanism network model. Second, regarding behavioral models, the antidepressant-like effects in the CUMS model, forced swimming test, and tail suspension test show some reproducibility, indicating its stable intervention potential in chronic stress-related depressive phenotypes. However, it should be noted that animal models can only partially mimic the pathological features of human depression, especially in terms of cognitive symptoms, the complexity of anhedonia, and individual differences. Therefore, existing animal data reflect more of an “anti-stress-like effect” than a complete equivalent to clinical antidepressant efficacy. Regarding clinical evidence, current research largely focuses on the observation of combination drugs or compound preparations. However, these studies generally suffer from small sample sizes, insufficient randomization and blinding, and short follow-up periods. The lack of rigorously designed, multicenter, double-blind, placebo-controlled randomized trials means that their clinical efficacy and safety cannot yet meet high-level evidence standards.

Elucidating the regulatory mechanisms by which important compounds in ZSS exert their antidepressant effects is beneficial for enriching germplasm resources and improving the quality of ZSS. However, the ZSS industry also faces many challenges. Ensuring the safety of ZSSn medicinal and edible products requires addressing pressing issues such as standardized cultivation, increasing yield and quality, and promoting the transformation of scientific research results. Solving these problems will lay the foundation for promoting the development of my country’s ZSS industry and economic growth. Only with strict quality standards, sufficient pharmacokinetic and safety data, and high-quality clinical evidence can ZSS move from traditional experience to evidence-based application, serving both as a standalone treatment/adjunctive therapy and as a functional dietary component, playing a unique role in overall population health management and mental health intervention. Therefore, future research can proceed in the following directions: First, establish a standardized quality control system to clarify the core biomarker components and their content ranges, thereby improving research reproducibility; second, conduct large-sample, multi-center, double-blind randomized controlled clinical trials to clarify its clinical positioning as a monotherapy or adjuvant therapy; third, strengthen pharmacokinetic and brain distribution studies, and construct a PK-PD association model; fourth, combine transcriptomics, metabolomics, and microbiome technologies to systematically analyze its multi-pathway synergistic regulatory mechanism; fifth, explore the possibility of precision medicine in the context of individual differences. While respecting the wisdom of traditional medicine, modern scientific methods should be used to transform the potential of ZSS into a widely applicable, monitorable, and truly beneficial intervention for public health.

## Glossary

CRH: Corticotropin-Releasing Hormone
WHO: World Health Organization
SSRIs: Selective Serotonin Reuptake Inhibitors
TCAs: Tricyclic Antidepressants
5-HT: 5-hydroxytryptamine
NE: Norepinephrine
DA: Dopamine
TrkB: Tyrosine Kinase receptor B
IL-6: Interleukin-6
IL-1: Interleukin-1
TNF-α: Tumor Necrosis Factor-alpha
CRP: C-reactive protein
LPS: Lipopolysaccharide
NF-kB: Nuclear Factor-k-gene Binding
IkBa: Inhibitor of Nuclear Factor-kB Alpha
TLR4: Toll-like receptor 4
SOD: Superoxide Dismutase
HPA: Hypothalamic-Pituitary-Adrenal
5-HIAA: 5-Hydroxyindole-3-acetic acid
cAMP: Cyclic Adenosine Monophosphate
MEK: Mitogen-Activated Protein Kinase Kinase
IL-8: Interleukin-8
IL-10: Interleukin-10
SYP: Synaptophysin
MyD88: Myeloid Differentiation Factor 88
HO1: Heme Oxygenase-1
NLRP3: NOD-like receptor pyrin domain containing 3
Nrf2: Nuclear Factor Erythroid-2-related Factor 2
HAMD: Hamilton Depression Scale
ACTH: Adrenocorticotropic Hormone
CORT: Cortisol
TCM: Traditional Chinese Medicine
JuA: Jujuboside A
JuB: Jujubosides B
JuC: Jujubosides C
CUMS: Chronic Unpredictable Mild Stress
GABA: γ-Aminobutyric Acid
Trp: Tryptophan
5-HTP: 5-hydroxytryptophan
ZO-1: Zonula Occludens-1
IL-1β: Interleukin-1β
BDNF: Brain-Derived Neurotrophic Factor
NO: Nitric Oxide
T-AOC: Total Antioxidant Capacity
GSH-Px: Glutathione peroxidase
CAT: Catalase
Glu: Glutamic Acid
GCs: Glucocorticoids
RSK: Ribosomal S6 Kinase
ECS: Endogenous Cannabinoids
IL-4: Interleukin-4
Hcy: Homocysteine
PSD-95: Postsynaptic Density Protein 95
GSDMD: Gasdermin-D
ERK: Extracellular Regulated Protein Kinases
CREB: cAMP response element bound protein
